# TULP2, a New RNA-Binding Protein, Is Required for Mouse Spermatid Differentiation and Male Fertility

**DOI:** 10.3389/fcell.2021.623738

**Published:** 2021-02-18

**Authors:** Meimei Zheng, Xu Chen, Yiqiang Cui, Wen Li, Haiqian Dai, Qiuling Yue, Hao Zhang, Ying Zheng, Xuejiang Guo, Hui Zhu

**Affiliations:** ^1^State Key Laboratory of Reproductive Medicine, Department of Histology and Embryology, Nanjing Medical University, Nanjing, China; ^2^Reproductive Medicine Center of No. 960 Hospital of PLA, Jinan, China; ^3^Department of Histology and Embryology, School of Medicine, Yangzhou University, Yangzhou, China

**Keywords:** spermatid differentiation, RNA binding protein, male infertility, oligo-astheno-teratozoospermia, TULP2

## Abstract

Spermatogenesis requires a large number of proteins to be properly expressed at certain stages, during which post-transcriptional regulation plays an important role. RNA-binding proteins (RBPs) are key players in post-transcriptional regulation, but only a few RBPs have been recognized and preliminary explored their function in spermatogenesis at present. Here we identified a new RBP tubby-like protein 2 (TULP2) and found three potential deleterious missense mutations of *Tulp2* gene in dyszoospermia patients. Therefore, we explored the function and mechanism of TULP2 in male reproduction. TULP2 was specifically expressed in the testis and localized to spermatids. Studies on *Tulp2* knockout mice demonstrated that the loss of TULP2 led to male sterility; on the one hand, increases in elongated spermatid apoptosis and restricted spermatid release resulted in a decreased sperm count; on the other hand, the abnormal differentiation of spermatids induced defective sperm tail structures and reduced ATP contents, influencing sperm motility. Transcriptome sequencing of mouse testis revealed the potential target molecular network of TULP2, which played its role in spermatogenesis by regulating specific transcripts related to the cytoskeleton, apoptosis, RNA metabolism and biosynthesis, and energy metabolism. We also explored the potential regulator of TULP2 protein function by using immunoprecipitation and mass spectrometry analysis, indicating that TUPL2 might be recognized by CCT8 and correctly folded by the CCT complex to play a role in spermiogenesis. Our results demonstrated the important role of TULP2 in spermatid differentiation and male fertility, which could provide an effective target for the clinical diagnosis and treatment of patients with oligo-astheno-teratozoospermia, and enrich the biological theory of the role of RBPs in male reproduction.

## Introduction

Approximately half of infertility is associated with the male partner (Salas-Huetos et al., 2017; Cao et al., 2018; Vander Borght and Wyns, 2018). Clinically, male infertility often manifests as azoospermia, oligozoospermia, teratozoospermia, and/or asthenozoospermia, resulting from underlying spermatogenesis disorders (Bracke et al., 2018). Therefore, research on the process and mechanism of spermatogenesis has been a hot spot in the field of reproductive medicine to reveal the pathogenesis of spermatogenic disorders and improve the diagnosis and treatment of male infertility.

Spermatogenesis is a complex process of cell division and differentiation, in which a large number of proteins expressed in a certain time sequence and a specific way participate or play a regulatory role (Chalmel and Rolland, 2015; Ozturk and Uysal, 2018). However, gene transcription is not continuous during spermatogenesis, and the differentiation of male germ cells involves long periods of transcriptional inactivation (one during the homologous recombination of spermatocytes entering early meiosis and another in late elongating spermatids at the time of chromatin condensation in spermiogenesis). Many mRNAs need to be synthesized in advance and stored at the ribonucleoprotein (RNP) granules called chromatoid bodies (Kotaja and Sassone-Corsi, 2007), then translated according to a certain time sequence (Paronetto and Sette, 2010). Thus, post-transcriptional regulation is particularly important for gene expression during spermatogenesis. Studies have shown that variable gene splicing occurs frequently in testicles, and at least 700 mRNAs are regulated at the translation level (Iguchi et al., 2006; Meijer et al., 2007; Grosso et al., 2008).

RNA-binding proteins (RBPs) are defined by their ability to recognize and bind to specific sequences of RNA, then regulate the fate of RNA, including the regulation of RNA surveillance, capping, splicing, nucleocytoplasmic transport to subcellular locations, translation and RNA degradation (Vanderweyde et al., 2013). Therefore, RBPs play a key role in the post-transcriptional regulation of gene expression. The role of RBPs in male reproduction has a few been reported. For example, DAZL can promote spermatogonia differentiation (Schrans-Stassen et al., 2001). The deficiency of PTBP2 results in increased spermatocyte apoptosis and stagnant differentiation of round spermatids (Zagore et al., 2015). Losing of TENR affects the count, morphology and motility of sperm (Connolly et al., 2005). All these indicate that RBPs play an important role in various stages of spermatogenesis and are key regulatory factors that ensure male fertility.

However, compared with the recognition of the importance of RBPs, there have been relatively few reports of the functions and mechanisms of RBPs. Only a small number of RBPs have been identified, and moreover, the biological functions and RNA targets of most known RBPs are still unclear. Therefore, screening RBPs during spermatogenesis and studying their functions and the molecular mechanism of their gene expression regulation after transcription will help to elucidate the molecular mechanism of spermatogenesis and provide valuable information related to the etiology, diagnosis and treatment of male infertility.

For this purpose, a mouse spermatogenic cell-specific RBP profile was generated in our laboratory with reference to the Kwon et al. (2013) method, and obtained many novel candidate RBPs (unpublished data). Among these, tubby-like protein 2 (TULP2) firstly attracted our attention. Mouse *Tulp2* has homologous genes in human that encode proteins belonging to the TULP family (North et al., 1997). Sequence analysis showed that both human and mouse TULP2 contained two conserved domains [Tub (Tubby) and DUF1168] but did not possess the canonical RBP domain (Albihlal and Gerber, 2018; Wang et al., 2018). However, with the discovery of new RBPs in recent years, it has been realized that the domains of RBPs may differ considerably from the original defined range, and a considerable number of RBPs without canonical RNA-binding domains (RBDs) are found *in vivo* (Ray et al., 2017). Mining and functional studies of these unconventional RBPs have become a focus of life science research (Beckmann et al., 2015). Similarly, this kind of RBPs may be more worthy of exploration in spermatogenesis.

Interestingly, there were the study examining the mRNA level of *Tulp2* in adult human multiple tissues and found it was only expressed in the testes, suggesting TULP2 might play an important role in male reproduction (North et al., 1997). Furthermore, by the analysis of *Tulp2* gene sequence on blood samples that we collected from 300 unrelated infertile men with dyszoospermia, we identified 13 cases of heterozygote (c.832C > T [p.Arg278Trp]) mutations, 2 cases of heterozygote (c.871A > G [p.Thr291Ala]) mutations, and 2 cases of heterozygote (c.829C > A [p.Leu277Met]) mutations. For these mutation sites, we searched Polyphen2 and SIFT databases and they were predicted to be potentially deleterious (Supplementary Figure 1). This result again indicated the potential relationship between *Tulp2* and male reproduction.

Therefore, we hypothesize that TULP2 is a new type of RBP in spermatogenic cells and play a role in male fertility. Further elucidation of its function may provide novel perspectives for the study of spermatogenesis mechanisms. In this study, a *Tulp2* knockout mouse model was constructed for the first time to explore the role and mechanism of TULP2. Based on the expression and localization characteristics, we focused on revealing the role of TULP2 in spermatid differentiation and its effect on sperm function, and initially elucidated the regulatory molecular network of TULP2. The results provide an effective target for the pathogenesis studies and the diagnosis and treatment of male infertility.

## Materials and Methods

### Animals

This study was approved by the ethics committees of Nanjing Medical University. *Tulp2* knockout mice were generated on the C57BL6 background via Cas9/RNA-mediated gene targeting as described previously (Shen et al., 2013) and bred at the animal center of Nanjing Medical University (Nanjing, China). The mice were kept under environmentally controlled conditions with unlimited access to food and water (Zhu et al., 2019). *Tulp2*^–/–^ mice were designed as the experimental group and littermate wild type (WT) mice as the control group.

### RNA Isolation, cDNA Synthesized, PCR and qPCR Analyses

Total RNA was extracted from tissues and using the TRIzol reagent (Invitrogen, 15596-026) according to the manufacturer’s instructions. cDNA was synthesized with PrimeScript RT reagent Kit (TaKaRa, RR037A). cDNA from multiple tissues of three adult WT mice and testes of 1–7 week-old WT mice (three mice of each age) were amplified by polymerase chain reaction (PCR) using random primers and Premix Taq (Takara, RR901). The PCR products were analyzed by 1.5% w/v agarose gel electrophoresis using mouse β-actin as the control gene. Quantitative real-time PCR of cDNA from testes of adult *Tulp2*^–/–^ mice and WT mice (three mice of each group) was performed using AceQ qPCR SYBR Green Master Mix (Vazyme, Q141-02) according to the manufacturer’s instructions with an ABI Q5 real-time PCR System (Applied Biosystems, Thermo Fisher Scientific). The primer sequences used for these experiments are listed in Supplementary Table 1.

### Western Blot Analysis

Protein samples were prepared from testes of *Tulp2*^–/–^ mice and WT mice (three mice of each group) or from 293T cells (three times cultured independently) using RIPA lysis buffer (Beyotime, P0013C) containing a protease inhibitor cocktail (Bimake, B14001). The samples were subjected to electrophoresis and transferred to nitrocellulose membranes (Bio-Rad, 1620177). The membranes were blocked and then incubated overnight with the following antibodies: anti-TULP2 (Abclonal produced the TULP2 antibody by the injection of rabbits with a peptide corresponding to amino acids 78–305 of the mouse protein; 1:5000), anti-CCT8 (Abclonal, A4449; 1:1000), anti-Flag (Proteintech, 20543-1-AP; 1:1000), and anti-ACTIN (Millipore, MAB1501; 1:8000). The membranes were then washed and incubated for 1 h with a horseradish peroxidase-conjugated anti-rabbit IgG secondary antibody (Thermo, 31460; 1:3000) or an anti-mouse IgG secondary antibody (Proteintech, SA00001-1; 1:1000). The proteins bands were detected using an ECL kit (Thermo, 32109) and Bio-Rad gel imaging system.

### Histological Analysis and Immunofluorescence

Histological analysis was performed by using standard procedures. Testes and epididymides from three *Tulp2*^–/–^ mice and three WT mice were fixed using modified Davidson’s fluid (Latendresse et al., 2002), and embedded in paraffin. The whole tissue was sectioned consecutively (5 μm thick) and collected on the slide. For the testis tissue, one section at an interval of 50 sections was taken for staining (total approximately 30 sections of each testis). Periodic acid-Schiff (PAS) staining was performed for testis sections according to the manufacturer’s protocol (Thermo, 87007) to determine the stage of spermatogenesis. Each stage showing a distinct ordering of cell associations along the length of the seminiferous tubules was designated with Roman numerals, and approximately 200 tubules from each section were analyzed under microscopy (Ahmed and de Rooij, 2009). According to our previously published protocols, epididymis sections (approximately four sections selected at intervals from each epididymis tissue) were stained with hematoxylin-eosin (HE) (Beyotime, C0105S) to observe sperm density (Zhu et al., 2019). Sperm from testis suspensions, the caput epididymis, the corpus epididymis, and the cauda epididymis of three *Tulp2*^–/–^ mice and three WT mice were transferred to slides (two sperm slides prepared from each tissue of each mouse) and naturally dried. After immobilization with 4% PFA, sperm morphology was observed by HE staining. The sections were viewed with an optical microscope (Zeiss, Germany). For ultrastructural examination, 2.5% glutaraldehyde-fixed sperm from cauda epididymis of three *Tulp2*^–/–^ mice and three WT mice were post-fixed with 2% (wt/vol) OsO4 and embedded in Araldite. Ultrathin sections were stained with uranyl acetate and lead citrate and analyzed using electron microscopy (JEOL, Japan).

For immunofluorescence analysis, the testicular section from each mouse was blocked in 1% bovine serum albumin and then incubated overnight at 4°C with primary antibodies against TULP2 and rabbit control IgG (Abclonal, AC005) at a dilution of 1:100. The sections were then incubated with a 488-conjugated secondary antibody (Proteintech, SA00013-2) at a 1:500 dilution for 1 h at room temperature. The slides were viewed with a LSM700 confocal microscope (Zeiss, Germany).

### Fertility Tests

Five adult *Tulp2*^–/–^ mice and WT mice (aged 2–3 months) were used for fertility study. Each male was mated with two females, and vaginal plugs were checked every morning. The number of offspring produced per female was recorded for 5 months.

### Computer-Assisted Sperm Analysis

Mature sperm from *Tulp2*^–/–^ and WT mice were obtained by making small incisions throughout the cauda epididymis, followed by extrusion and suspension in 200 μl of human tubal fluid (HTF) culture medium (EasyCheck, M1130). The sperm samples (10 μl) were subjected to Computer Assisted Sperm Analyzer (CASA) detection with the IVOS II^TM^ system (Hamilton Thorne, United States). Motility parameters for three couple mice (the experimental and control groups) were measured and analyzed.

### Terminal Deoxynucleotidyl Transferase dUTP Nick End Labeling Assays

DNA fragmentation was determined as an index of apoptosis in paraffin-embedded mouse testis sections from three *Tulp2*^–/–^ mice and three WT mice via the terminal deoxynucleotidyl transferase dUTP nick end labeling (TUNEL) assay (Vazyme, A113-01) according to the manufacturer’s specifications. Briefly, the testis sections were washed in PBS and equilibrated with TdT buffer for 20 min at room temperature. TdT buffer was removed and terminal transferase reaction mix was added. The reaction was performed for 1 h at 37°C. Sections were washed with PBS and counterstained with Hoechst. All spermatogenesis tubules (approximately 200 tubules) from each section from each mouse were analyzed using a LSM700 confocal microscope (Zeiss, Germany). The nuclei of TUNEL-positive cells show red fluorescence. According to the stage of spermatogenesis and the nuclear morphology of spermatogenic cells, apoptotic cells were classified and counted by two researchers using double-blind.

### Measurement of Sperm ATP *in vitro*

Sperm samples from three *Tulp2*^–/–^ mice and three WT mice were washed twice and resuspended in lysis buffer, then vortexed and placed on ice. An average of 5 × 10^7^ sperm were used for ATP analysis. ATP was measured by luminometric methods using commercially available luciferin/luciferase reagents as per the manufacturer’s instructions (Beyotime, S0027) in a luminometer (TD-20/20, Turner Designs).

### Immunoprecipitation and Mass Spectrometry

HEK293T cells were transfected with the Flag-TULP2 overexpression plasmid using Lipofectamine 3000 (Thermo Fisher Scientific), and empty vector plasmid was used as control. 48 h after transfection, cells were collected for immunoprecipitation via a protocol that was described previously (Zhu et al., 2019). Cell transfection and immunoprecipitation experiments were performed three times. Then the immunoprecipitates were analyzed by mass spectrometry as described previously (Zhu et al., 2019). In brief, elution was first performed in protein extraction buffer by ultrafiltration using a 10-kDa filter membrane. Cysteine residues were reduced with dithiothreitol (DTT) at a final concentration of 5 mmol/L at 56°C for 25 min and then alkylated in iodoacetamide at 14 mmol/L for 30 min at room temperature. Unreacted iodoacetamide was quenched with DTT for 15 min. The lysates were then diluted to 1.6 mol/L urea with 25 mmol/L Tris at pH 8.2. CaCl_2_ was added to a final concentration of 1 mmol/L, followed by overnight digestion with trypsin at a concentration of 5 ng/μL at 37°C. Finally, 0.4% trifluoroacetic acid was added to stop digestion. We used an OASIS HLB extraction box (Waters, Milford, MA, United States) to desalinate the peptides, followed by mass spectrometry analysis. All raw files were searched using MaxQuant software (version 1.3.0.5, Martinsried, Germany) against the UniProt human proteome database.

### Co-immunoprecipitation

Co-immunoprecipitation was performed as described previously (Zhu et al., 2010). Testes from three WT mice were lysed using RIPA buffer supplemented with a 1% (v/v) protease inhibitor cocktail instead of lysis buffer. The lysate was incubated on ice for 40 min, followed by centrifugation at 12000 × *g* for 20 min at 4°C. The supernatant was pre-cleared with 50 μl of Protein A/G magnetic beads (Bimake, B23202) and then incubated with the target antibody at 4°C overnight. A normal IgG antibody was used as a control. The next day, the co-immunoprecipitated proteins were eluted with Pierce IgG Elution Buffer (Thermo, 21004), followed by western blotting.

### Transcriptome Sequencing Analysis

Total testis RNA was extracted respectively from three wild-type and three *Tulp2*^–/–^ mice using TRIzol reagent (Invitrogen), followed by clean up with an RNeasy mini kit (QIAGEN, 74104), and RNA quality was examined. A total amount of 1 μg of qualified RNA per sample was used as the input material for library preparation. The libraries were generated using the VAHTS mRNA-seq v2 Library Prep Kit for Illumina (Vazyme, NR601) following the manufacturer’s recommendations. The library concentration was measured using a Qubit RNA Assay Kit on a Qubit 3.0 fluorometer for preliminary quantification. After it was verified that the insert size was consistent with expectations, the qualified insert size was accurately quantified using qPCR with a Step One Plus Real-Time PCR system (ABI, United States). The clustering of the index-coded samples was performed on a cBot Cluster Generation System (Illumia, United States) according to the manufacturer’s instructions. After cluster generation, the library preparations were sequenced on the Illumina HiSeq × Ten platform and 150 bp paired-end module. The uniquely mapped reads of genes were counted using DESeq2, and the presence of transcripts abundances were expressed as fragments per kilobase of exon per million fragments mapped (FPKM). Based on the FPKM values of WT mice, the transcripts were normalized to evaluate the differences in gene expression of *Tulp2*^–/–^ mice. Significant differentially expressed genes were screened according to a fold change >1.5, and the DAVID database was used to analyze Gene Ontology (GO) annotations.

### Statistical Analysis

All experiments were repeated at least three times. The differences between the treatment and control groups were analyzed using a *t*-test. Values of *P* < 0.05 were considered to be statistically significant. All data represent the mean ± the standard deviation of the mean (SD).

## Results

### TULP2 Is Specifically Expressed in the Testis and Localized to Spermatids

**FIGURE 1:**
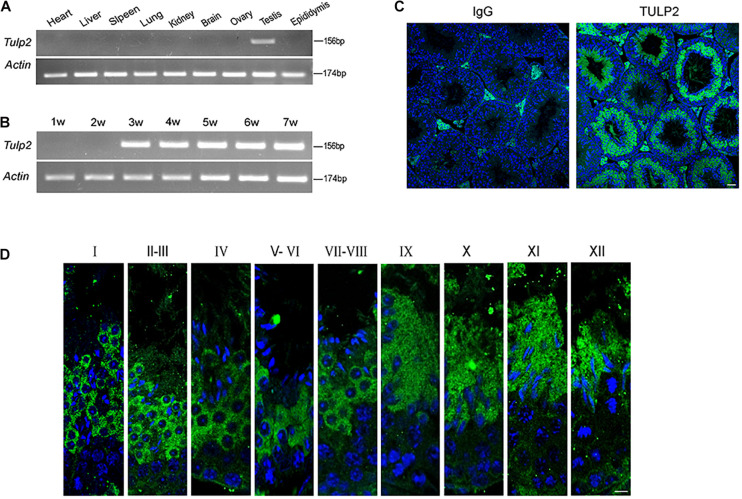
Distribution of of TULP2 in mouse tissues. **(A)** The presence of *Tulp2* mRNA was evaluated in various tissues from adult mice using RT-PCR. *Tulp2* mRNA was only detected in the testis. *Actin* was amplified as an internal control. **(B)** The testicular *Tulp2* mRNA expression profile was tested at the indicated time points after birth using RT-PCR. *Actin* was amplified as an internal control. *Tulp2* expression was first observed in 3-week-old mice and continued until adulthood. **(C)** Immunofluorescence localization of the TULP2 protein in adult mouse testes. TULP2 expression in germ cells within the seminiferous tubules. **(D)** Each image shows a stage of the seminiferous epithelial cycle, denoted by Roman numerals at the top of each image. Anti-TULP2 antibody (green) and Hoechst staining (blue). TULP2 was expressed in the cytoplasm of step 1–12 spermatids in spermiogenesis. Scale bars: 20 μm.

Reverse transcription polymerase chain reaction (RT-PCR) was used to evaluate the distribution of *Tulp2* mRNA in various tissues of mice, including the heart, liver, spleen, lung, kidney, brain, ovary, testis, and epididymis. As shown in Figure 1A, *Tulp2* was only expressed in the testis. Testis maturation is age dependent, and the first wave of spermatogenesis takes place within 35 days postnatal in mice (Griswold, 2016). Analysis using testis mRNA obtained from mice of different ages in the weeks after birth showed that *Tulp2* was first detected in the testes of 3-week-old mice, when round spermatids had appeared, after which its expression remained constant (Figure 1B). We further detected the expression characteristics of TULP2 in the testis by immunofluorescence (IF) analysis. The results demonstrated TULP2 expression in germ cells within the seminiferous tubules (Figure 1C); its expression was initiated in round spermatids and remained until step 12 elongated spermatids (Figure 1D). The above results suggested a potential important role of TULP2 in male reproductive function, very likely influencing spermiogenesis in the testis.

### Targeted Disruption of TULP2 Results in Male Infertility

**FIGURE 2:**
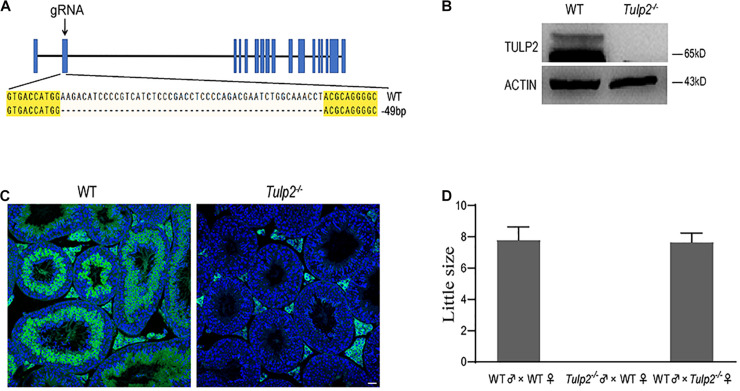
Generation of *Tulp2* knockout mice and assessment of male fertility. **(A)** Image of Sanger sequencing results showing a 49-bp deletion in the targeted region. **(B)** Western blot analysis of testicular TULP2 expression. ACTIN was used as an internal control. TULP2 was detected in WT mice, whereas it was absent in *Tulp2*^−/−^ mice. **(C)** TULP2 levels in the testes of *Tulp2*^−/−^ and WT mice were evaluated via immunofluorescence analysis. No TULP2 signal was detected in *Tulp2*^−/−^ mice. Scale bars: 20 μm. **(D)** Average litter size of pups produced by *Tulp2*^−/−^ mice mated with WT mice. *Tulp2*^−/−^ male mice are infertile (*n* = 5).

To investigate whether TULP2 is required for male fertility, *Tulp2* knockout mice were produced using the CRISPR/Cas9 system. The injection of a single guide RNA targeting *Tulp2* exon 2 produced knockout mice harboring a 49-bp deletion (Figure 2A). A representative image of Sanger sequence results for the verification of the *Tulp2*^–/–^ mouse is shown in Figure 2A. We confirmed the lack of TULP2 in the testes of *Tulp2*^–/–^ mice by western blot and immunofluorescence assays (Figures 2B,C).

Mating tests were performed, and continuous monitoring was conducted for 5 months to analyze the fertility of the mice. As a result, we found that the female *Tulp2*^–/–^ mice were fertile and exhibited litter sizes comparable to those of WT females. In contrast, *Tulp2*^–/–^ males were infertile and produced no litters (Figure 2D). These results suggest that TULP2 is required for male reproduction.

### Decreases in the Sperm Count and Motility Are the Potential Cause of Infertility in *Tulp2*^–/–^ Male Mice

**FIGURE 3:**
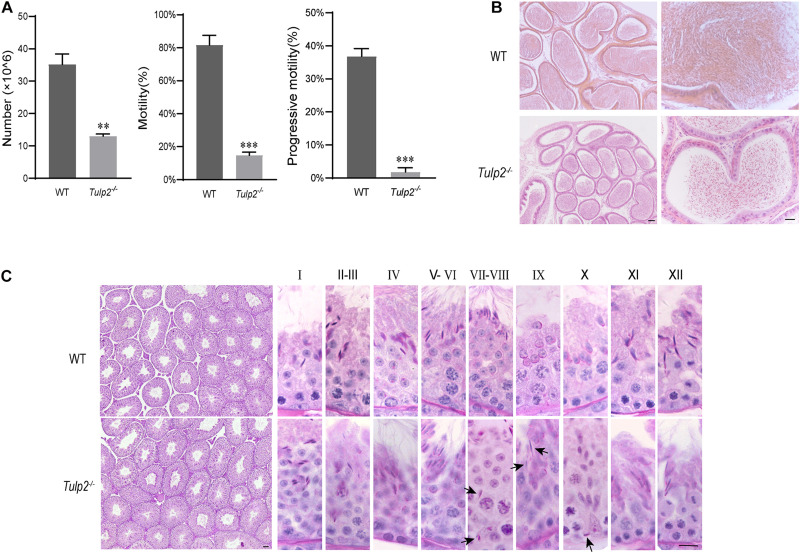
Analysis of basic factors related to male fertility. **(A)** Computer-assisted sperm analysis. The sperm number was reduced, and motility and progressive motility were decreased in *Tulp2*^−/−^ mice (*n* = 3, ^∗∗^ *P* < 0.01, ^∗∗∗^*P* < 0.001). **(B)** HE-stained sections show the histological structure of the epididymis from WT and *Tulp2*^−/−^ mice. The number of sperm in the *Tulp2*^−/−^ mouse epididymis was decreased compared with that in WT mice. **(C)** Periodic acid-Schiff-stained sections show the histological structure of the testes from WT and *Tulp2*^−/−^ mice. Each image shows a stage of the seminiferous epithelial cycle, which is denoted by Roman numerals at the top of the image. The spermatogenic lumen is complete, and the spermatogenic cells are arranged neatly. There are residual elongated spermatids in the lumen of stage VII–X (black arrow). Scale bars: 50 μm.

To determine the cause of infertility in male *Tulp2*^–/–^ mice, we examined the physiological parameters of sperm collected from cauda epididymis of the mice. CASA showed that the sperm count, motility and progressive motility were significantly decreased in *Tulp2*^–/–^ mice compared with WT mice (Figure 3A, ^∗∗^*P* < 0.01, ^∗∗∗^*P* < 0.001). The decreased sperm number was verified by the assessment of the histological structure of the epididymis. As shown in Figure 3B, sperm quantities in the lumen of the epididymis duct were obviously reduced in *Tulp2*^–/–^ mice compared with WT mice.

### Increased Apoptosis and Limited Release of Elongated Spermatids Reduce the Epididymis Sperm Count in *Tulp2*^–/–^ Mice

Sperm are produced in the seminiferous tubules of the testis. To determine the cause of the observed decrease in sperm output, we first observed the histological structure of the testis by morphological staining. Intact spermatogenic tubules and spermatogenic cells at all stages could be seen in the testes of WT and *Tulp2*^–/–^ mice. However, retained elongated spermatids were frequently observed in the middle and base of stage VII–X seminiferous tubules of *Tulp2*^–/–^ mice, which should have been released into the lumen at stage VIII (Figure 3C, black arrow). This may be responsible for the decreased number of epididymal sperm in *Tulp2*^–/–^ mice.

**FIGURE 4:**
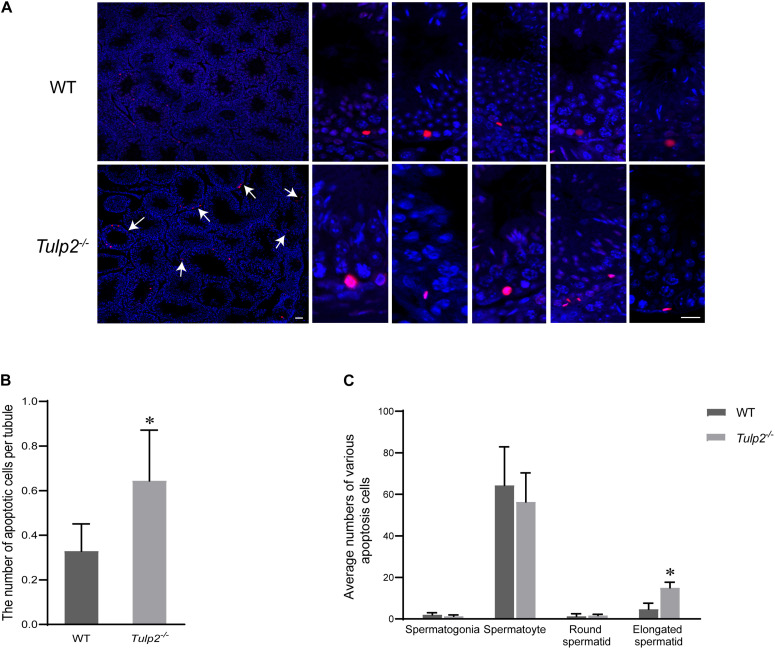
Analysis of the causes of spermatozoa loss. **(A)** Statistics of testicular apoptosis. Terminal deoxynucleotidyl transferase nick-end-labeling (TUNEL) staining in testes. The apoptotic cells in *Tulp2*^−/−^ mice were significantly increased (as shown by white arrow). The TUNEL-positive cells show red fluorescence localization in the nucleus. Scale bars: 50 μm. **(B)** Analysis of the number of apoptotic cells per tubule. The number of cells was increased in *Tulp2*^−/−^ mice (*n* = 3, ^∗^*P* < 0.05). **(C)** Average numbers of various apoptotic cells were classified and counted. The number of elongated spermatids showing apoptosis increased significantly (*n* = 3, ^∗^*P* < 0.05).

To reveal the reason for limited elongated spermatid release, we evaluated the state of spermatogenic cells in *Tulp2*^–/–^ and WT mice. TUNEL analysis was performed in the testes of these two groups, and the results showed that the average number of apoptotic cells per tubule was significantly increased in *Tulp2*^–/–^ mice (Figure 4B, ^∗^*P* < 0.05). According to the classified statistics, the apoptosis of elongated spermatids in *Tulp2*^–/–^ mice was significantly increased compared with that in WT mice (Figure 4C, ^∗^*P* < 0.05), and these apoptotic cells were mainly elongated spermatids deposited in the base of the lumen (Figure 4A).

### Abnormal Differentiation of Spermatids Results in a Defective Sperm Tail, Which Decreases Sperm Motility in *Tulp2*^–/–^ Mice

**FIGURE 5:**
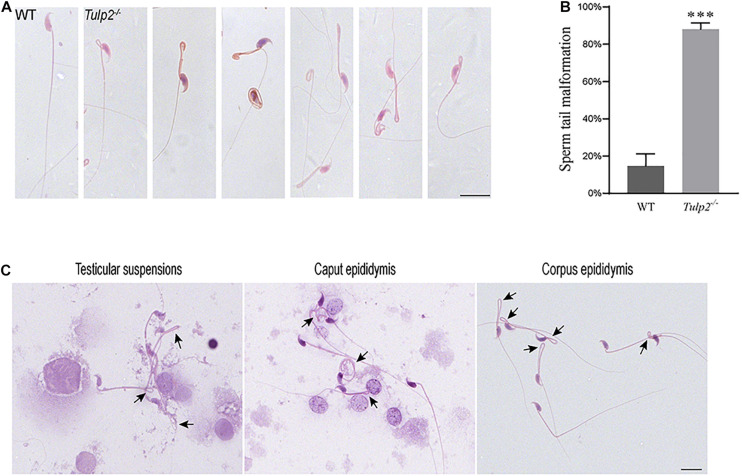
Analysis of sperm malformation. **(A)** The morphology of spermatozoa in WT and *Tulp2*^−/−^ mice. The sperm of *Tulp2*^−/−^ mice showed tail folding and curling. **(B)** Statistics tail malformation rates. Tail malformation was significantly increased in *Tulp2*^−/−^ mice (*n* = 3, ^∗∗∗^*P* < 0.001). **(C)** Spermatozoon morphology was observed in testis suspensions, the caput epididymis and the corpus epididymis. The black arrow showed sperm tail malformation. Scale bars: 20 μm.

We observed the morphology and structure of spermatozoa from the cauda epididymis. Under light microscopy, the spermatozoa from WT mice showed a normal morphology, including well-shaped heads and long, smooth tails. However, obvious tail abnormalities were found in the sperm of *Tulp2*^–/–^ mice, mainly exhibiting tail folding and curling (Figure 5A). According to the statistics, the deformity rate of the sperm tail in *Tulp2*^–/–^ mice was significantly higher than that in WT mice (Figure 5B, ^∗∗∗^*P* < 0.001). By observing the spermatozoa in testicular suspensions, the caput epididymis and the corpus epididymis from *Tulp2*^–/–^ mice, we found that they showed deformities consistent with the sperm in the cauda epididymis (Figure 5C). The above results indicated that sperm deformity originated from the abnormal differentiation of spermatids and that the loss of TULP2 affected spermiogenesis in the testes.

**FIGURE 6:**
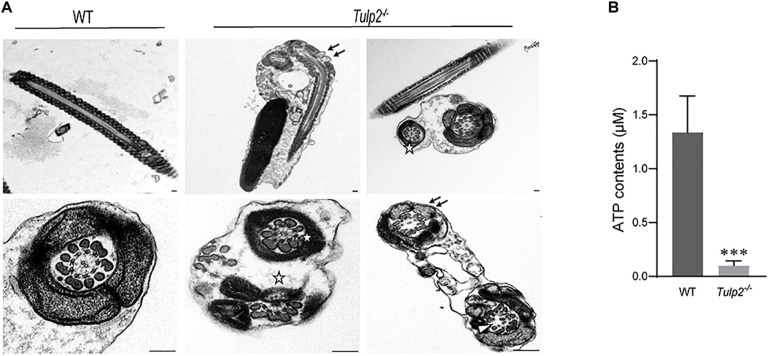
Assessment of sperm ultrastructure and ATP levels. **(A)** Transmission electron microscopy (TEM) images of sperm ultrastructures are shown, revealing a lack of inner mitochondrial membrane (IMM) cristae (as shown by “↑”), partial absence of “9 + 2” microtubules (as shown by “Δ”), and incomplete peripheral dense fibers (as shown by “✩”) (*n* = 3). Scale bars: 1 μm. **(B)** Tested ATP contents of sperm between WT and *Tulp2*^−/−^ mice. The ATP level was significantly lower in *Tulp2*^−/−^ mice (*n* = 3, ^∗∗∗^*P* < 0.001).

The ultrastructure of sperm from the two groups was further observed under a transmission electron microscope. As expected, sperm from the WT mice showed normally developed flagella. However, an obviously increase in structural abnormalities of the sperm tail was found in *Tulp2*^–/–^ mice. In these mice, two or more cross-sections of the same sperm flagellum were frequently found enclosed in one cell membrane; in the flagellum, mitochondria, microtubules and peripheral dense fibers were all structurally abnormal, which mainly manifested as a lack of inner mitochondrial membrane (IMM) cristae (as shown by “↑”), partial absence of “9 + 2” microtubules (as shown “Δ”), and incomplete peripheral dense fibers (as shown “✩”) (Figure 6A).

Mitochondria, peripheral dense fibers and microtubules are the structural basis of sperm motility, and abnormalities of these structures inevitably affect sperm motility. Among these structures, the mitochondrial sheath is the center of the sperm energy supply. We detected the ATP content of the sperm and found that it was significantly lower in *Tulp2*^–/–^ mice than in wild-type mice (Figure 6B, ^∗∗∗^*P* < 0.001). All of the above results suggest that TULP2 deletion may affect the process of spermiogenesis and result in damage to mitochondria, microtubules, and peripheral dense fibers, which are responsible for decreased sperm motility.

### The Absence of TULP2 Affects the Expression of a Number of Genes in Mouse Testes

**FIGURE 7:**
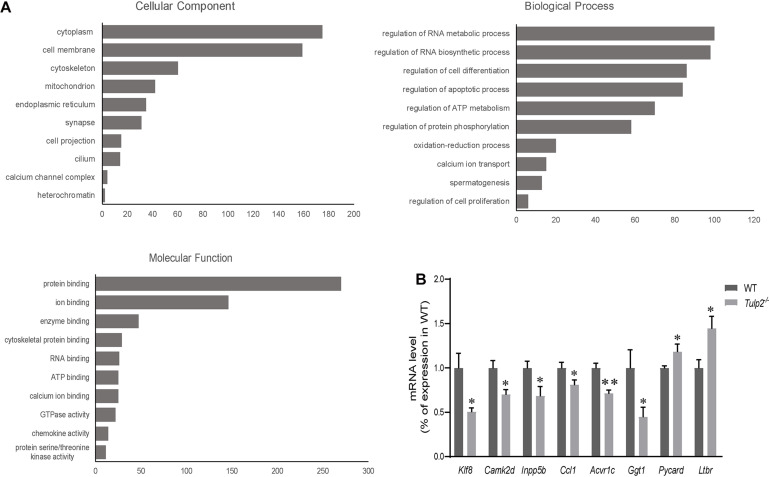
Analysis of mRNA transcriptome sequencing data. **(A)** Genes showing a difference of more than 1.5 fold were analyzed according to GO annotations and classified into three major categories: cellular component, biological process, and molecular function. **(B)** The genes in each pathway were verified using real-time PCR. The results were consistent with the sequencing data (*n* = 3, ^∗^*P* < 0.05, ^∗∗^*P* < 0.01).

As an RBP, TULP2 should play its role in spermatogenesis by regulating specific target RNAs. To further analyze and clarify the mechanism by which TULP2 affects spermatogenesis, the differential expression profiles of testicular transcripts in *Tulp2*^–/–^ and WT mice were established by transcriptome sequencing. As a result, 1446 genes with an expression difference of 1.5 fold or more were identified between the two groups (Supplementary Table 2), among which 638 presented GO annotations. The GO annotations were classified into three major categories: cellular components, biological processes and molecular functions (Figure 7A). In the category of cellular components, most of the differentially expressed genes were most strongly associated with the cytoplasm, followed by the plasma membrane, cytoskeleton or mitochondrion, and a few were related to components such as cilia or projections. The classification according to biological processes indicated that the differentially expressed genes were mostly related to the regulation of RNA metabolism and biosynthesis, followed by cell events (apoptosis and differentiation), while others were involved in energy metabolism, spermatogenesis, etc. When we analyzed molecular functions, we found that these genes mainly showed binding activity, including the binding of proteins, cytoskeletal, enzymes, RNA, ATP, ions, etc.

According to the results of GO annotation, we selected genes related to key events to test their expression levels to verify the sequencing results. The apoptosis-related genes included *Pycard* (PYD and CARD domain containing) and *Ltbr* (lymphotoxin B receptor). The energy metabolism-related genes included *Ccl1* [chemokine (C-C motif) ligand 1], *Camk2d* (calcium/calmodulin-dependent protein kinase type II, delta), and *Acvr1c* (activin A receptor, type 1C). The spermatogenesis-related genes included *Inpp5b* (inositol polyphosphate-5-phosphatase B) and *Ggt1* (gamma-glutamyl transferase 1). The RNA metabolism-related gene *Klf8* (kruppel-like factor 8) was also included. The real-time PCR results were consistent with the sequencing data (Figure 7B, ^∗^*P* < 0.05, ^∗∗^*P* < 0.01).

### TULP2 Interacts With CCT8, Participating in the Regulation of Spermatogenesis

**FIGURE 8:**
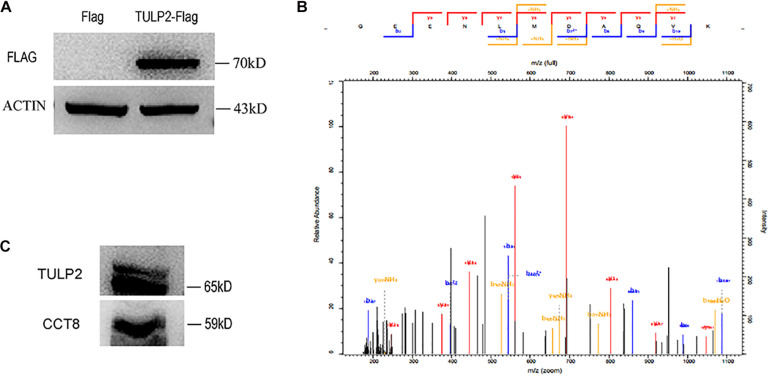
The discovery of TULP2-interacting protein. **(A)** Flag-TULP2 overexpression plasmid was transfected into the HEK293T cell line and successfully expressed. **(B)** The cellular immunoprecipitates were analyzed by mass spectrometry and identified the CCT8. **(C)** Co-immunoprecipitation assays were performed using anti-TULP2 and anti-CCT8 antibodies in WT mice testis. TULP2 was detectable in immunoprecipitates prepared from the testes of WT mice using antibodies against CCT8. CCT8 was detectable in immunoprecipitates prepared from the testes of WT mice using the anti-TULP2 antibody.

To further investigate the molecular function of TULP2, we constructed a TULP2 overexpression plasmid with Flag label and transfected it into the HEK293T cell line. After the transfection of the TULP2 plasmid, the cellular proteins were extracted, and the transfection efficiency was detected by western blotting with an anti-Flag antibody. The results showed that the TULP2 protein was successfully expressed in HEK293T cells (Figure 8A). HEK293T cell proteins were extracted after transfection with the TULP2 plasmid, and an immunoprecipitation experiment was carried out. Then, the samples were identified using mass spectrometry, which detected CCT8 (T-complex protein 1 subunit theta) (Figure 8B). Co-immunoprecipitation and western blot analysis using anti-TULP2 and anti-CCT8 antibodies were performed to examine the interaction between TULP2 and CCT8. TULP2 was detectable in immunoprecipitates prepared from the testes of WT mice using antibodies against CCT8. Similarly, CCT8 was detectable in immunoprecipitates prepared from the testes of WT mice using the anti-TULP2 antibody (Figure 8C).

## Discussion

Spermatogenesis takes place in the testis and is one of the most complex differentiation events that occur within developmental biology, necessitating the controlled regulation of gene expression (Sutherland et al., 2015). Post-transcriptional regulation of gene expression is essential for the progression of spermatogenesis (Idler and Yan, 2012). Thus, the study of RBPs, which is the core factors in post-transcriptional regulation, has become a key focus in the field of male reproductive biology.

Based on our previous study, we focused on a new candidate RNA binding protein, TULP2, and through cross-linking immunoprecipitation (CLIP) confirming that TULP2 could indeed bind RNAs (Supplementary Figure 2). The exact role of TULP2 in spermatogenesis and male reproduction is still unknown. In this study, we constructed *Tulp2* gene knockout mice to study the effect of the absence of the product of this gene. The results confirmed that TULP2 is necessary for maintaining male fertility and *Tulp2*^–/–^ mice showed the phenotype of oligo-astheno-teratozoospermia. In these mice, abnormal deposition of apoptotic elongated spermatids in testis was observed (resulting the decreased sperm count in epididymis); the sperm showed reduced motility and tail deformity, in which the structures of microtubule, mitochondria and peripheral dense fibers were all damaged.

The sufficient quantity and quality of sperm is the guarantee of male fertility, which depends on the orderly completion of spermatogenesis (Neto et al., 2016). We confirm the specific expression of TULP2 in testicular spermatid, which suggests that TULP2 is involved in spermatid differentiation during spermatogenesis. In the process of spermatid differentiation, round spermatids undergo dramatic morphological, molecular and cellular alterations, laying a foundation for the generation of functional sperm (Ozturk et al., 2017; Zakrzewski et al., 2021). Fully developed elongated spermatids ultimate release from the seminiferous epithelium into the tubule lumen to ensure an adequate sperm count (Wen et al., 2016). Defects at various structure of spermatid during spermiogenesis may cause elongated spermatid to be retained within the epithelium and apoptosis (O’Donnell, 2015). In addition, abnormal differentiation of spermatid may result in structural defects of sperm, decreased motility, and impaired fertilization (Ray et al., 2017). Therefore, our results confirmed that TULP2 is a key factor in regulating spermatid differentiation and ensuring sperm quality.

As an RBP, TULP2 theoretically plays a role in post-transcriptional regulation and affects the expression of a series of genes, resulting in various phenotypes (Idler and Yan, 2012; Kang et al., 2020). The analysis of these downstream genes could furtherly elucidate the molecular mechanism of TULP2 regulating spermatogenesis. We established the differential expression profiles of testicular transcripts between *Tulp2*^–/–^ and WT mice by transcriptome sequencing, and 1446 genes exhibiting an expression difference of more than 1.5 fold were identified. These differentially expressed transcripts should be direct or indirect targets of TULP2, and also the molecules that TULP2 affects when it plays the role. By using the bioinformatics analysis (GO annotation), we furtherly elucidate the association between the exertion of TULP2 function and these molecular changes.

According to the analysis of cellular components, these differentially expressed genes were mainly related to the cytoplasm, especially structures such as the cytoskeleton, mitochondria, and cilia. This characteristics was consistent with the function that TULP2 participates in the differentiation of spermatids. During spermiogenesis, the structures and components of the cytoplasm of spermatids undergo major alterations and transformations (Ozturk et al., 2017; Zakrzewski et al., 2021). Structural differentiation in the cytoplasm includes processes such as the development of the Golgi apparatus into the acrosome and the extension and assembly of the flagellum (Lehti and Sironen, 2016; Touré et al., 2020). The key structures in the sperm flagellum related to motility include the cytoskeleton, mitochondria, etc. (Pleuger et al., 2020), which were the categories showing the highest abundance of differentially expressed genes.

Based on the analysis of biological processes, most of the differentially expressed genes were associated with the regulation of RNA metabolism and biosynthesis; a further literature review indicated that these genes mainly consisted of transcription factors and that some were RBPs (Xing et al., 2019; Hardison et al., 2020) (Supplementary Figure 3A). Both of transcription factors and RBPs regulate the downstream target molecular network to control a plethora of developmental and physiological processes (Hentze et al., 2018; Auer et al., 2020). Therefore, we think that TULP2 is a relatively upstream regulatory factor, which indirectly confirms the important role of TULP2 in spermatogenesis. In addition, many differentially expressed genes were associated with biological processes such as apoptosis, energy metabolism and spermatogenesis. Such molecular changes correspond to the results of our phenotype research, which confirm that TULP2 uses these molecules to perform its function.

From the perspective of molecular functions, the differentially expressed genes were mainly related to binding activity, particularly protein binding (including cytoskeletal proteins and enzymes) (Supplementary Figure 3B), and the literature review showed these enzymes are mainly related to energy metabolism (Matsumoto et al., 2012; Cuvelier et al., 2018). We think this should be related to the role of TULP2 in the differentiation of the sperm tail. The most important components of the sperm flagellum are cytoskeletal proteins (Lehti and Sironen, 2017), while enzyme activity is the molecular basis for ATP generation and sperm motility (Moraes and Meyers, 2018). Thus, TULP2 regulates the expression of these genes to participate in the assembly of the sperm tail and energy metabolism. On the other hand, “protein binding” activity may be due to the considerable number of transcription factors among the potential targets that are regulated by TULP2, which usually bind related co-transcription factors when performing their functions (Herzel et al., 2017). In addition, some potential targets of TULP2 showed RNA-binding activity, which are assumed to be the RBPs that we have analyzed in the “biological process” section.

Therefore, the GO analysis verified the role of TULP2 in spermatid differentiation from different perspectives, and the results of the biological process and molecular function analyses also confirmed each other. To gain a deeper understanding of the molecular function of TULP2 in spermiogenesis, we showed that TULP2 interacts with CTT8 by mass spectrometry and co-immunoprecipitation. CCT8 is a subunit of the CCT complex (also known as the TCP1 complex) (Brackley and Grantham, 2009). As a cytosolic chaperonin, the CCT complex assists in the folding of approximately 10% of cytosolic proteins to achieve their native structure (Jin et al., 2019; Vallin and Grantham, 2019). Studies in planarian flatworms suggest that each CCT subunit may play important roles in spermiogenesis (Counts et al., 2017). Therefore, we speculate that TULP2 might be the substrate of CCT8 and be correctly folded by the CCT complex, which requires further verification in the future.

In summary, TULP2 is a new RBP that we identified for the first time that is specifically expressed in the testes and localized to spermatids. We demonstrated that TULP2 participates in the normal differentiation of spermatids by regulating a series of transcripts related to the cytoskeleton, mitochondria and apoptosis and thereby affects sperm counts and tail assembly. In this process, CCT8 may bind to TULP2, which is correctly folded by the CCT complex and plays a role in spermiogenesis (schematic diagram for the role of TULP2 in spermiogenesis was shown in Supplementary Figure 4). Due to the typical oligo-astheno-teratozoospermia and infertility phenotype exhibited by mice after the loss of TULP2, and three potential deleterious missense mutations of this gene had been found in dyszoospermia patients, *Tulp2* is likely to be a potential pathogenic gene in patients with these conditions. TULP2 and its downstream molecules become the potential targets for the clinical diagnosis and treatment of these patients. In the future, further research is required to confirm if potential deleterious mutations affect TULP2 protein function.

## Data Availability Statement

The datasets presented in this study can be found in online repositories. The names of the repository/repositories and accession number(s) can be found in the article/Supplementary Material.

## Ethics Statement

The animal study was reviewed and approved by Ethics Committee of Experimental Animal Welfare, Nanjing Medical University.

## Author Contributions

HuZ and XG conceived and designed the project. YC, MZ, and QY performed the experiments. MZ, XC, and YC collected the data. MZ, XC, WL, HD, HaZ, HuZ, and YZ analyzed the data. MZ and XC created the figures and tables and drafted the manuscript. HuZ critically revised the manuscript. All authors read and approved the final version of the manuscript.

## Conflict of Interest

The authors declare that the research was conducted in the absence of any commercial or financial relationships that could be construed as a potential conflict of interest.

## Supplementary Material

Supplementary Figure 1 Identification of three potential deleterious missense mutations of *Tulp2* gene in dyszoospermia patients.

Supplementary Figure 2 TULP2 binds RNAs through cross-linking immunoprecipitation. **(A)** TULP2 plasmid with Flag label was transfected into HEK293T cells. The HEK293T cell proteins were extracted after transfection, and an immunoprecipitation experiment was carried out. **(B)** The autoradiograph of a CLIP experiment revealed TULP2 can bind RNA (black arrow).

Supplementary Figure 3 The classification and proportions of differentially expressed genes were summarized according to the results of GO analysis. **(A)** The classification and proportions of differentially expressed genes related to the regulation of RNA metabolism and biosynthesis, and mainly were transcription factors and some were RBPs. **(B)** The classification and proportions of differentially expressed genes related to the protein binding, mainly were transcription factors, enzymes and cytoskeletal proteins.

Supplementary Figure 4 Schematic diagram for the role of TULP2 in spermiogenesis. TULP2 interact with CCT8 and regulate a series of transcripts to play a role in normal spermatid differentiation. The loss of TULP2 leads to abnormal spermatids differentiation, increased spermatids apoptosis and abnormal spermatids deposition, resulting in reduced sperm count, decreased motility and sperm malformation.

Supplementary Table 1 Primer sequences and target fragment size of each gene.

Supplementary Table 2 1446 genes showing an expression difference of 1.5 fold or more.
