# Supplementary figures and images for: TULP2, a New RNA-Binding Protein, Is Required for Mouse Spermatid Differentiation and Male Fertility
Source: Front Cell Dev Biol. 2021 Feb 18;9:623738. doi: 10.3389/fcell.2021.623738 (PMC7982829; doi:10.3389/fcell.2021.623738)

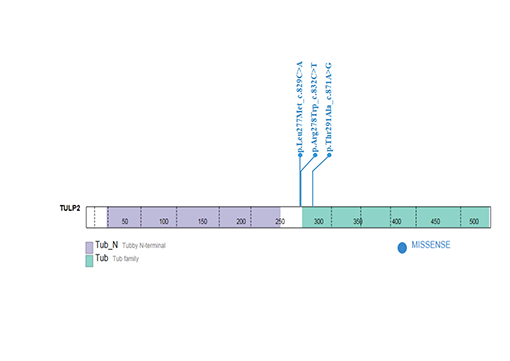

Supplement: Supplementary Figure 1 — Identification of three potential deleterious missense mutations of Tulp2 gene in dyszoospermia patients. [file Image_1.TIFF]

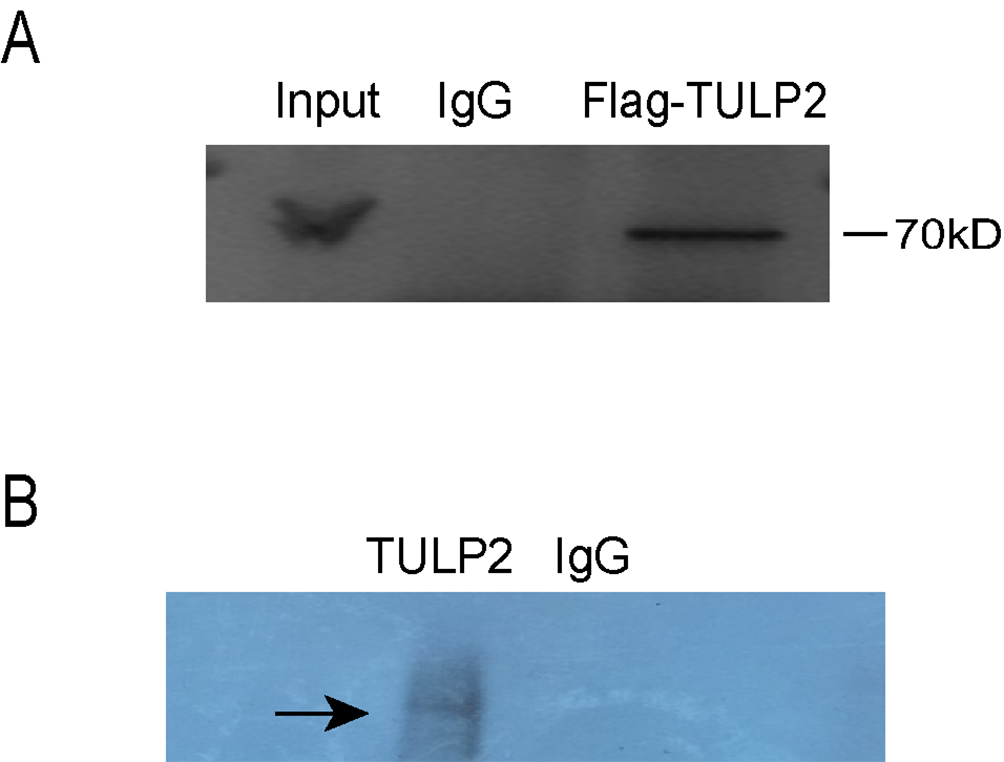

Supplement: Supplementary Figure 2 — TULP2 binds RNAs through cross-linking immunoprecipitation. (A) TULP2 plasmid with Flag label was transfected into HEK293T cells. The HEK293T cell proteins were extracted after transfection, and an immunoprecipitation experiment was carried out. (B) The autoradiograph of a CLIP experiment revealed TULP2 can bind RNA (black arrow). [file Image_2.TIF]

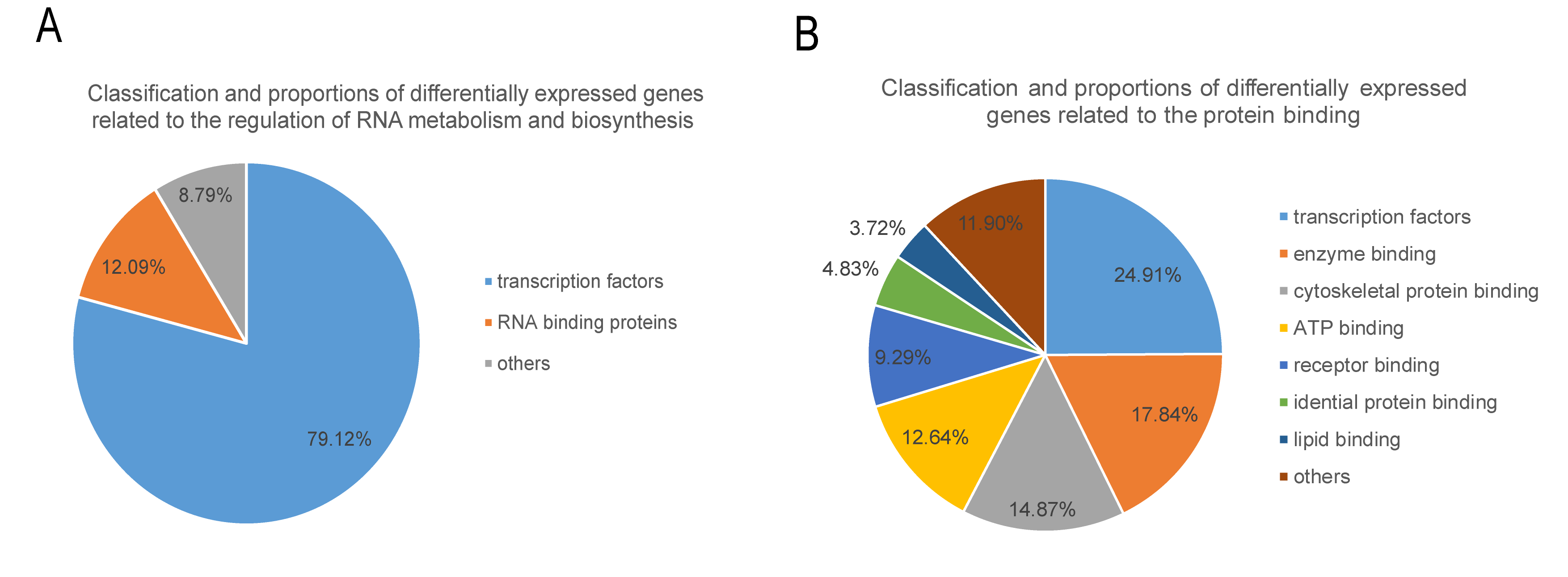

Supplement: Supplementary Figure 3 — The classification and proportions of differentially expressed genes were summarized according to the results of GO analysis. (A) The classification and proportions of differentially expressed genes related to the regulation of RNA metabolism and biosynthesis, and mainly were transcription factors and some were RBPs. (B) The classification and proportions of differentially expressed genes related to the protein binding, mainly were transcription factors, enzymes and cytoskeletal proteins. [file Image_3.tif]

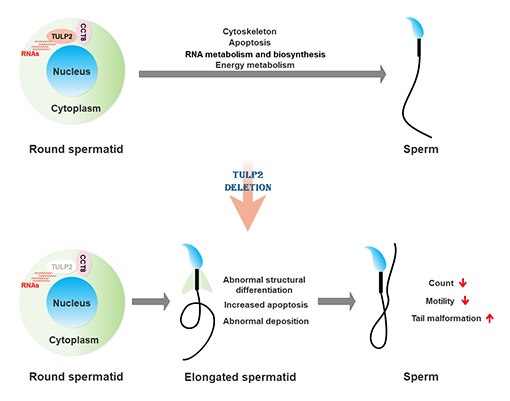

Supplement: Supplementary Figure 4 — Schematic diagram for the role of TULP2 in spermiogenesis. TULP2 interact with CCT8 and regulate a series of transcripts to play a role in normal spermatid differentiation. The loss of TULP2 leads to abnormal spermatids differentiation, increased spermatids apoptosis and abnormal spermatids deposition, resulting in reduced sperm count, decreased motility and sperm malformation. [file Image_4.TIFF]
